# Interferon-γ augments GABA release in the developing neocortex *via* nitric oxide synthase/soluble guanylate cyclase and constrains network activity

**DOI:** 10.3389/fncel.2022.913299

**Published:** 2022-08-10

**Authors:** Noah Döhne, Alice Falck, Gabriel M. S. Janach, Egor Byvaltcev, Ulf Strauss

**Affiliations:** ^1^Institute of Cell Biology and Neurobiology, Charité—Universitätsmedizin Berlin, Corporate Member of Freie Universität Berlin, Humboldt-Universität zu Berlin, Berlin, Germany; ^2^Institute of Neuroscience, Lobachevsky State, University of Nizhny Novgorod, Nizhny Novgorod, Russia

**Keywords:** IFN-γ, type-II interferon, cerebral cortex, neuroimmunology, phasic inhibition, MEA, NO

## Abstract

Interferon-γ (IFN-γ), a cytokine with neuromodulatory properties, has been shown to enhance inhibitory transmission. Because early inhibitory neurotransmission sculpts functional neuronal circuits, its developmental alteration may have grave consequences. Here, we investigated the acute effects of IFN-γ on γ-amino-butyric acid (GABA)ergic currents in layer 5 pyramidal neurons of the somatosensory cortex of rats at the end of the first postnatal week, a period of GABA-dependent cortical maturation. IFN-γ acutely increased the frequency and amplitude of spontaneous/miniature inhibitory postsynaptic currents (s/mIPSC), and this could not be reversed within 30 min. Neither the increase in amplitude nor frequency of IPSCs was due to upregulated interneuron excitability as revealed by current clamp recordings of layer 5 interneurons labeled with VGAT-Venus in transgenic rats. As we previously reported in more mature animals, IPSC amplitude increase upon IFN-γ activity was dependent on postsynaptic protein kinase C (PKC), indicating a similar activating mechanism. Unlike augmented IPSC amplitude, however, we did not consistently observe an increased IPSC frequency in our previous studies on more mature animals. Focusing on increased IPSC frequency, we have now identified a different activating mechanism—one that is independent of postsynaptic PKC but is dependent on inducible nitric oxide synthase (iNOS) and soluble guanylate cyclase (sGC). In addition, IFN-γ shifted short-term synaptic plasticity toward facilitation as revealed by a paired-pulse paradigm. The latter change in presynaptic function was not reproduced by the application of a nitric oxide donor. Functionally, IFN-γ-mediated alterations in GABAergic transmission overall constrained early neocortical activity in a partly nitric oxide–dependent manner as revealed by microelectrode array field recordings in brain slices analyzed with a spike-sorting algorithm. In summary, with IFN-γ-induced, NO-dependent augmentation of spontaneous GABA release, we have here identified a mechanism by which inflammation in the central nervous system (CNS) plausibly modulates neuronal development.

## Introduction

IFN-γ, the only type-II IFN, is a pleiotropic inflammatory cytokine and contributes to immune defense against viruses, bacteria, and tumors (Kak et al., 2018). As a part of the adaptive and innate immune response, IFN-γ is mainly secreted by lymphocytes and functionally acts by influencing gene expression, protein synthesis, and induction of an antiproliferative state on most cell types by activating IFN-γ receptor and subsequently the JAK-STAT1 (Janus kinase–signal transducer and activator of transcription 1) pathway (Ivashkiv, 2018). IFN-γ is present in central nervous system (CNS) at baseline levels and is elevated in conditions, such as viral infections, CNS trauma, and cerebral ischemia (Monteiro et al., 2017). In the CNS, IFN-γ serves an important neuron-specific function (Clark et al., 2022), notably by enabling non-cytolytic viral clearance (Patterson et al., 2002).

Beyond its canonical role in controlling intracellular pathogens, a growing body of evidence suggests a direct impact of IFN-γ on the CNS. The absence of IFN-γ leads to neurodevelopmental abnormalities (Monteiro et al., 2015) that differ in healthy vs. inflammatory conditions (Litteljohn et al., 2014). Notably, some neuropsychiatric diseases, such as autism spectrum disorder (ASD; Saghazadeh et al., 2019) and depression (Schmidt et al., 2014), are associated with increased IFN-γ levels. Developmental disturbances due to withdrawal of care (i.e., maternal deprivation) also cause elevated IFN-γ levels as shown in hippocampus and prefrontal cortex of newborn rats (Giridharan et al., 2019) and intrauterine inflammation leads to the accumulation of IFN-γ-producing γ/δ T cells in the brain of fetal mice (Lewis et al., 2021). However, T cells of patients with schizophrenia exhibit decreased production of IFN-γ (Arolt et al., 2000), and the IFN-γ inducer Anaferon improved outcomes in schizophrenia therapy (Vetlugina et al., 2016). As many neuropsychiatric conditions display altered IFN-γ levels, and IFN-γ knockout produces changes in behavior and cognitive performance (for review Monteiro et al., 2017), taken together extant evidence indicates a possible role for IFN-γ in maintaining healthy brain function.

Moreover, IFN-γ has been reported to have fast-acting neuromodulatory properties (Müller et al., 1993; Janach et al., 2022). IFN-γ attenuates *I*_h_, but unlike type-I IFNs (Stadler et al., 2014), it leaves pyramidal neurons’ excitability unchanged due to acutely augmented inhibition. In particular, IFN-γ augments GABA (γ-amino-butyric acid)ergic currents in the developed rat neocortex (Janach et al., 2020) by increasing synaptic GABA_*A*_ channel number in a protein kinase C (PKC)-dependent manner (Janach et al., 2022). In line with this, IFN-γ augments GABAergic currents in the hippocampus (Brask et al., 2004; Flood et al., 2019).

Such alterations of electrical properties could particularly affect neuronal development that is tightly regulated by early neuronal activity in addition to genetic fate (Kirischuk et al., 2017). With regard to the latter, GABA (in particular) plays a key role (Le Magueresse and Monyer, 2013). For instance, on the one hand, GABA acts excitatory during early neurodevelopment due to elevated intracellular chloride (Ben-Ari, 2002), and during development (excitatory) GABA regulates glutamatergic synapse formation *via N*-methyl-D-aspartate receptor (NMDA) activation (Wang and Kriegstein, 2008). On the other hand, GABA may also inhibit neocortical neurons in the first postnatal week (Daw et al., 2007). However, GABAergic neurons also coordinate the development of local cortical networks during a narrow critical postnatal period (Modol et al., 2020). Given these few selected examples of findings concerning the established role of GABA in neuronal development, it does not come as a surprise that the disruption of developing GABAergic neocortical inhibitory network has been implicated in neurodevelopmental disorders, including schizophrenia (Lewis et al., 2005), epilepsy (Cobos et al., 2005), and autism (Pizzarelli and Cherubini, 2011). If IFN-γ altered GABAergic transmission in the developing brain, then this would have broad neurodevelopmental implications, including modulation of cortical maturation.

Taken together, it is clear that the developing brain is vulnerable to disruption of GABAergic transmission, IFN-γ can alter GABAergic currents, and IFN-γ is present at a baseline level or might be elevated by several conditions in the developing brain. Unraveling interactions of the immune system and developing neuronal circuits will foster a better understanding of the biology behind neurodevelopmental diseases. In the long run, such understanding might enable the treatment or even prevention of neurodevelopmental diseases. As a first step, we have here addressed on both, a single cell and a network level, whether IFN-γ affects inhibitory transmission in neocortical neuronal development, and if so whether the effect is comparable to the one we have previously observed in more mature animals. We have focused on GABAergic synapses at a critical developmental stage (P6–7) during ongoing GABAergic synaptogenesis in rats (Luhmann and Prince, 1991). We mainly investigated spontaneous events because they play a crucial role in synaptic structure and function and act autonomously in regulating synaptic plasticity and homeostasis (Kavalali, 2015).

## Materials and methods

### Interferon-γ

Chinese hamster ovary or *E. coli*-derived recombinant IFN-γ (U-Cytech, Utrecht, Netherlands; Active Bioscience, Hamburg, Germany) was reconstituted in double distilled water, aliquoted to 1.0 e^5^ IU, and stored at −20°C. Aliquots were thawed directly prior to usage. Final concentration for all experiments was 1,000 IU ml^–1^.

### Animals

For all experiments, Wistar pups of either sex were used on postnatal days 6 and 7 (P6–7). All experiments were carried out in agreement with the European Communities Council Directive of 22 September 2010 (2010/63/EU) under the licenses T 0212/14, T-CH 0034/20 for wild-type rats, and T0215/11 for transgenic rats. For some experiments, we used W-Tg(Slc32a1-YFP*)1Yyan vesicular GABA transporter (VGAT)-Venus transgenic rats that express the yellow fluorescent protein Venus in VGAT expressing neurons, developed by Uematsu et al. (2008) that enabled us to identify interneurons in the slice.

### Acute slice preparation

As generally described in Zaqout et al. (2019), decapitation was followed by rapid removal of the brain, which then was transferred into carbogenated (95% O_2_; 5% CO_2_) 2°C sucrose artificial cerebrospinal fluid (sACSF) containing (in mM): 85 NaCl, 2.5 KCl, 1 NaH_2_PO_4_, 7 MgCl_2_, 26 NaHCO_3_, 50 sucrose, 10 D(+)-glucose (Carl Roth, Karlsruhe, Germany), and 0.5 CaCl_2_ (Merck, Darmstadt, Germany). 300 μM thick coronary slice containing somatosensory cortex were cut using a VT1200S vibratome (Leica, Wetzlar, Germany). Slices were recovered for 30 min in 34°C sACSF and were subsequently held at room temperature in modified ACSF containing (in mM): 92 NaCl, 2.5 KCl, 1.2 NaH_2_PO_4_, 30 NaHCO_3_, 25 D(+)-glucose (Carl Roth), 5 sodium ascorbate, 20 2-[4-(2-hydroxyethyl)piperazin-1-yl] ethanesulfonic acid (HEPES), 3 sodium pyruvate, 2 MgSO_4_, 2 CaCl_2_ (Merck), and 2 thiourea (VWR Chemicals, Radnor, PA, United States) until recording. All variations of ACSF were continuously carbogenated.

### *Ex vivo* patch clamp recordings

Slices were transferred to a submerged type recording chamber continuously perfused with carbogenated ACSF containing (in mM): 119 NaCl, 2.5 KCl, 1 NaH_2_PO_4_, 1.3 MgCl_2_, 26 NaHCO_3_, 10 D(+)-glucose (Carl Roth), and 2.5 CaCl_2_ (Merck) at 32 ± 2°C. Slices were visualized using infrared differential interference contrast (DIC) microscopy with a Zeiss Axioskop 2 FS plus (Carl Zeiss, Oberkochen, Germany). Glass micropipettes (2–5 MΩ) were pulled from lead (PG10165-4, WPI, Sarasota, FL, United States) or borosilicate (GB150-10P, Science Products, Hofheim, Germany) glass capillaries using a P-97 micropipette puller (Sutter Instrument, Novato, CA, United States). For IPSC recordings, 6-cyano-7-nitroquinoxaline-2,3-dione (CNQX, 20 μM) and D(-)-2-Amino-5-phosphonopentanoic acid (DAP-5, 25 μM) (Tocris, Bristol, United Kingdom) were added to the perfusate to block glutamatergic ion currents and pipettes were filled with a high chloride internal solution containing (in mM): 140 CsCl (Biomol, Hamburg, Germany), 4 NaCl, 1 MgCl_2_ (Carl Roth), 10 HEPES, 0.1 ethylene glycol-bis(β-aminoethyl ether)-N,N,N’,N’-tetraacetic acid (EGTA), 0.3 GTP, 2 Mg^2+–^ATP (Merck), (305 mOsm, pH set to 7.25 with KOH), and 5 mM QX-314 (Merck) to improve the signal-to-noise ratio. Calphostin C (Enzo Life Sciences, NY, United States) was dissolved in DMSO at 1 mM and aliquoted to working sizes. Prior to respective experiments, Calphostin C was added to the pipette solution to a final concentration of 100 nM. When Calphostin C was used, slice and pipette were continuously exposed to full-spectrum white light (Bruns et al., 1991). Other drugs in this study included: Bicuculline methiodide (Tocris), *S*-Nitroso-*N*-acetylpenicillamine (SNAP) (cayman chemical, Ann Arbor, MI, United States), 1H-[1,2,4]oxadiazolo[4,3-a]quinoxalin-1-one (ODQ) (Tocris), tetrodotoxin (TTX) (Tocris), and *N*-[3(Aminomethyl)benzyl] acetamidine dihydrochloride (1400W) (Sigma-Aldrich, St. Louis, United States). ODQ was dissolved in dimethylsulfoxide (DMSO) at 100 mM and aliquoted to working sizes. Total DMSO in perfusate never exceeded 0.1%. All other reagents and drugs, unless specified otherwise, were dissolved and aliquoted in double distilled water.

For current-clamp recordings, internal solution contained (in mM) 120 K-Gluconate, 11 EGTA, 10 HEPES, 10 phosphocreatine, 1 MgCl_2_, 2 Mg^2+^-ATP, 0.3 GTP, 10 KCl (Merck), and 1 CaCl_2_ (Carl Roth). After establishing whole-cell configuration, pipette solution was allowed to diffuse into the cell, and blockers were bath applied for at least 5 min before recording started. Spontaneous/miniature inhibitory postsynaptic current (s/mIPSC) recordings were excluded from analysis when series resistance (*R*_s_) changed by more than 25%, initial IPSC frequency was below 10 events min^–1^ (0.167 events s^–1^), holding current (that was mainly due Cl^–^ enhanced tonic inhibition and Cs^+^ mediated depolarization, Supplementary Figure 1A) changed abruptly or hyperpolarized beyond −800 pA, indicating the development of technical leak. If access resistance changed during experiments, efforts were made to reestablish access, and experiments were discontinued if unsuccessful. IFN-γ exposure time was aimed at 25 min. In some cases, a later time point (up to 30 min) or an earlier time point (minimum 15 min) was used. Reasons included changed series resistance that had to be corrected or sudden changes in holding current. An overview of exposure times is given in Supplementary Figure 1B, population data averages are stated in the respective figure legends. Electric activity was recorded using an EPC-10 double amplifier (HEKA Elektronik GmbH, Reutlingen, Germany), digitized at 6.25 kHz after Bessel filtering (2.5 kHz), and stored *via* PatchMaster 2.91 (HEKA). Spontaneous and miniature IPSCs were analyzed using Synaptosoft MiniAnalysis Software (Synaptosoft Inc., Fort Lee, NJ, United States). Negative deflections deviating from baseline by more than 3 RMS (root mean square) noise were counted as IPSCs. Upon manual review, erroneously detected events were removed and missed events were added. For every data point, events from two consecutive minutes of recordings were averaged. For washout experiments, slices were incubated in ACSF containing IFN-γ, CNQX (20 μM), and DAP-5 (25 μM) at 34°C for 30 min. IPSCs were recorded under continuous perfusion of the same solution until stable recording conditions were achieved. Then, IFN-γ was removed from the perfusion. Electrically evoked orthodromic IPSCs have been rarely demonstrated before P14 (Luhmann and Prince, 1991). In this study, stimulating currents (0.02–1 mA, applied for 100 μs, delivered through a 1 MΩ glass electrode filled with ACSF, positioned 150 μm apically and 200 μm laterally of the recorded neuron) reliably evoked delayed inward current responses that could be blocked by bicuculline, confirming their GABAergic identity (Supplementary Figure 2E). To avoid stimulus-induced long-term changes in synaptic transmission, paired stimuli were applied at 10 s intervals. For paired-pulse experiments, *R*_s_ changes up to 35% were tolerated. Paired-pulse recordings were analyzed using FitMaster v2 × 90.5 (HEKA). For every datapoint, 20 traces were analyzed by dividing the amplitude of the second-evoked IPSC through the amplitude of the first. Of 20 ratios, the mean ratio was calculated for comparison. Current-clamp recordings were analyzed using FitMaster software for spike detection and counting. Calculations requiring polynomial fitting were performed using OriginPro 2022 (OriginLab, Northhampton, MA, United States). Neurons recorded in voltage clamp had a mean capacitance of *C*_before_ = 118.0 ± 3.4 pF that slightly decreased to *C*_after_ = 112.0 ± 3.2 pF (*p* < 0.0001, *n* = 111, *N* = 42 paired *t*-test) over the course of voltage clamp recordings.

### *Post hoc* visualization

For cell selection, pyramidal neurons were identified by their typical morphology in DIC microscopy, VGAT expressing interneurons could be identified *via* fluorescence. To validate correct cell selection, in a subset of recordings, N_ε_ -(+)-Biotinyl-L-lysine (biocytin) (Invitrogen, Waltham, MA, Unites States) was added to the pipette solution to a final concentration of 1%. After recording, slices were fixed in cold phosphate-buffered saline with 4% paraformaldehyde for 1 h. Then, slices were washed in 0.1 M phosphate buffer and subsequently incubated in 4°C phosphate buffer with 0.3% Triton-X (Sigma-Aldrich), 0.05% NaN_3_ (Merck), and fluorescent-conjugated streptavidin (Alexa Fluor 647, 1:1,000, Invitrogen) for 48 h. Following another washing step, slices were mounted and imaged using a laser scanning confocal microscope (FV1000, Olympus, Tokyo, Japan).

### Multielectrode array recordings

Neuronal activity was recorded with a Multielectrode Array (MEA), consisting of 60 electrodes in an 8 × 8 layout with electrode diameters of 10 μM, and 200 μM spacing (60MEA200/10iR, Multi Channel Systems (MCS), Reutlingen, Germany). Slices, prepared as described above, were placed in the recording chamber, so that somatosensory cortex covered the electrodes. The recording chamber was briefly drained to ensure contact between slice surface and electrodes. Slices were secured by steel anchors (Warner Instruments, Holliston, United States) and were continually perfused with carbogenated ACSF (4–6 ml min^–1^), maintained at 30°C by the Pt100 Temperature Controller System (MCS). In all recordings, the K^+^-channel blocker 4-Aminopyridine (50 μM, 4-AP, Sigma-Aldrich) was used to enhance activity. Recordings were started after 5 min of stabilization and lasted 30 min, under control and test condition, respectively. Electrical activity was recorded by the MEA 2100-120 system (bandwidth = 1–3,000 Hz, gain = × 10, MCS) and sampled at 10 kHz using Multi Channel Experimenter software (MCS). Given the electrode distance of 200 μm, it is unlikely but possible that events cross the detection threshold on multiple channels. Event sorting was performed with the spyKing circus algorithm (Yger et al., 2018) to avoid repetitive counting. Event detection threshold was set to 8 MAD (mean absolute deviation) from baseline of a given channel. Data were high-pass filtered at 300 Hz (3rd-order Butterworth filter) and whitened using the spyKing circus software.^1^ Event sorting was performed over the entire recording, including control and test conditions. Sorted data were reviewed with PHY graphical user interface.^2^ For further analysis, we included the last 20 min of each condition. Only event clusters with normally distributed amplitudes were considered for analysis.

### Statistical analysis and data reporting

The study was designed to enable longitudinal pairwise comparison. Note that a direct comparison of IPSC frequencies between different neurons is hampered by the huge variance of the frequency of GABAergic inputs at this developmental stage that might be due to staggered maturation, and many other factors (for instance slicing angle, depth in the slice, quality of individual preparation, Supplementary Figures 2A–C). All statistical analyses were performed using OriginPro 2022 (OriginLab). Datasets were tested for normal distribution using the Shapiro–Wilk test. In case of rejection (*p* ≤ 0.05), the Wilcoxon signed-rank test for paired data was used to test for statistical significance. Probability distributions were tested for equality using the Kolmogorov–Smirnov test. Due to low event counts, data for all recordings under a certain experimental condition were pooled for probability distributions. Total number of events analyzed, number of recordings, and number of animals are given in the respective figure legends. When normal distribution could not be rejected, Student’s *t*-test or paired Student’s *t*-test was used, respectively. The null hypothesis was rejected for *p* ≤ 0.05. All data are given in *d*_variable_ = mean ± standard error of the mean (*P*, statistical test, *n* = number of recordings, *N* = number of animals), unless otherwise specified.

### Figures and images

Figures were plotted using OriginPro 2022 (OriginLab). Images of biocytin-filled neurons were adjusted (brightness inversion, contrast, scale bars, z-stack projection) with Fiji software (Schindelin et al., 2012). Final assembly was achieved using Corel Draw 2017 (Corel Corporation, Ottawa, ON, Canada).

## Results

### Interferon-γ acutely increased sIPSC frequency and amplitude

**FIGURE 1:**
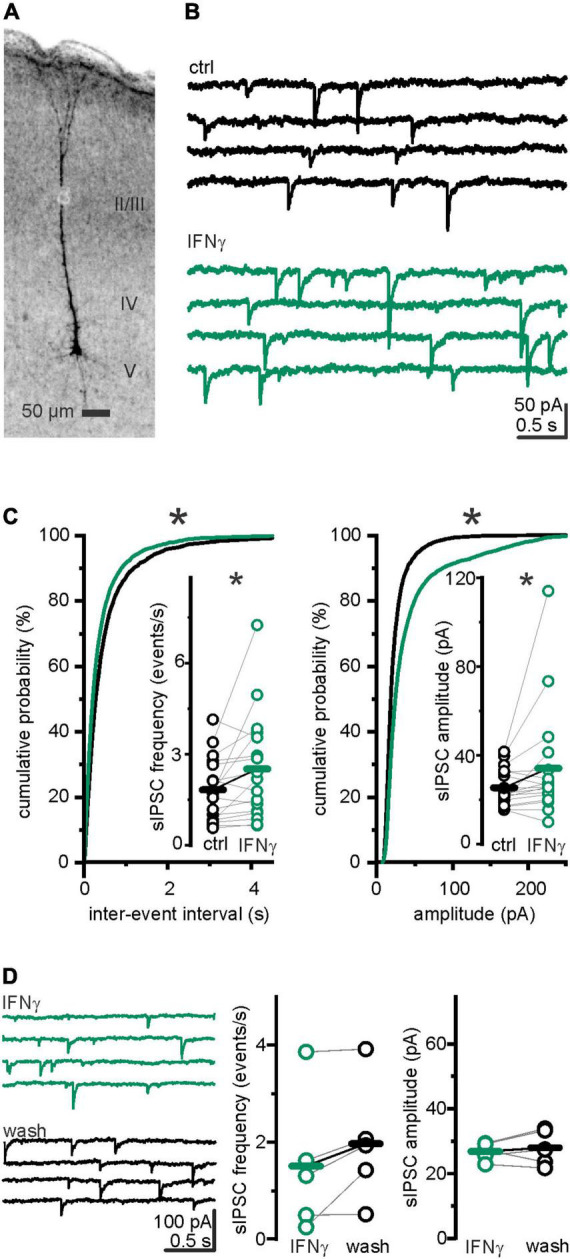
IFN-γ increased frequency and amplitude of spontaneous IPSCs in layer 5 pyramidal neurons of P6–7 rats. **(A)** Example image of a biocytin-filled neuron ensuring its layer 5 pyramidal identity. Brightness inverted, z-stack projection at average intensity. **(B)** Current traces with spontaneous IPSCs (sIPSCs) recorded before (control) and under IFN-γ (1,000 IU ml^–1^). **(C)** Cumulative probability plots of all amplitudes and inter-event intervals (IEI) before (*black*) and under (*green*) IFN-γ application. IFN-γ application shifted the amplitude distribution curve to higher amplitudes (*p* < 0.0001; Kolmogorov–Smirnov test, ctrl = 3,671 events vs. IFN-γ = 5,108 events, *n* = 17 recordings, *N* = 8) and shorter IEIs (*p* < 0.0001; Kolmogorov–Smirnov test, ctrl = 3,637 IEIs vs. IFN-γ = 5,074 IEIs, *n* = 17 recordings, *N* = 8). Population data reveal increased sIPSC frequency and amplitude upon IFN-γ (23.1 ± 0.8 min) (*inset*). **(D)** sIPSC amplitude and frequency remained stable for at least 30 min after IFN-γ was removed from the perfusate, as example traces (*left*) and population data (*right*) show. Series resistance (*R*_s_) slightly increased from *R*_s ctrl_ = 11.1 ± 1.3 MΩ to *R*_s IFN–γ_ = 11.9 ± 1.5 MΩ (*p* = 0.02, paired Wilcoxon signed-rank test, *n* = 17, *N* = 8). Holding current was reduced from *I*_ctrl_ = −243.2 ± 39.6 pA to *I*_IFN–γ_ = −153.3 ± 26.6 pA (*p* = 0.02, paired Wilcoxon signed-rank test, *n* = 17, *N* = 8). Elevated *R*_s_ cannot cause IPSC amplitude or frequency increase. During washout experiments, series resistance slightly increased from *R*_s IFN–γ_ = 14.6 ± 2.1 MΩ to *R*_*s* wash_ = 16.4 ± 2.4 MΩ (*p* = 0.04, paired *t*-test, *n* = 5, *N* = 1). Holding current remained comparable (*I*_hold IFN–γ_ = −144.0 ± 26.1 pA vs. *I*_hold wash_ = −121.9 ± 20.0 pA; *p* = 0.3, paired *t*-test, *n* = 5, *N* = 1). *p < 0.05.

We have previously found an increased inhibition in the neocortex of late juvenile and adult rats under the influence of IFN-γ (Janach et al., 2020). Given the prominent role of GABAergic transmission in cortical development, herein, we have studied IFN-γ effects on developing neurons. In detail, we whole-cell recorded spontaneous inhibitory postsynaptic currents (sIPSCs) in somatosensory pyramidal layer 5 neurons (Figure 1A) from P6 to 7 Wistar rats. As seen in more mature neurons, bath-applied IFN-γ (1,000 IU ml^–1^) increased the mean amplitude of sIPSCs at P6–7 from *I*_ctrl_ = 25.6 ± 2.2 pA to *I*_IFN–γ_ = 36.1 ± 6.7 pA (*p* = 0.005, Wilcoxon signed-rank test, *n* = 17, *N* = 8; Figures 1B,C). Surprisingly, the sIPSC frequency also increased markedly from *f*_ctrl_ = 1.96 ± 0.27 events s^–1^ to *f*_IFN–γ_ = 2.73 ± 0.46 events s^–1^ (*p* = 0.039, Wilcoxon signed-rank test, *n* = 17, *N* = 8). We then addressed whether these relatively acute effects ceased upon removal of IFN-γ. We found that, when preincubated with IFN-γ for at least 30 min, sIPSC frequency and amplitude remained stable for at least 30 min during washout (*f*_IFN–γ_ = 1.51 ± 0.64 events s^–1^ vs. *f*_wash_ = 1.97 ± 0.56 events s^–1^, *p* = 0.09, paired *t*-test, *n* = 5, *N* = 1; *I*_IFN–γ_ = 26.8 ± 1.2 pA vs. *I*_wash_ = 28.0 ± 2.5 pA, *p* = 0.5, paired *t*-test, *n* = 5, *N* = 1; Figure 1D). This indicates that IFN-γ induced changes in GABAergic transmission do not vanish within 30 min. Instead, the trend toward higher frequencies plausibly indicates an inductive effect of IFN-γ on sIPSC frequency. To test whether our intracellular solution produced changes in sIPSC frequency or amplitude during long-term experiments (i.e., if this is merely a time-dependent effect), we recorded sIPSCs for 15–20 min in plain ACSF and found that both, frequency (*f*_before_ = 2.4 ± 0.3 events s^–1^ vs. *f*_after_ = 2.5 ± 0.4 events s^–1^; *p* = 0.62, paired *t*-test, *n* = 11, *N* = 3) and amplitude (*I*_before_ = 27.5 ± 3.1 pA vs. *I*_after_ = 24.3 ± 2.2 pA; *p* = 0.19, paired *t*-test, *n* = 11, *N* = 3), remained comparable (Supplementary Figure 1C). The considerable increase in sIPSC frequency may suggest a presynaptic action in addition to the previously described postsynaptic mechanism (Janach et al., 2022) behind the increased amplitude. Therewith, the observed augmentation of inhibition by IFN-γ is not only reproduced in the developing rat brain but exhibits a feature that is not consistently observed at later stages of brain development.

### Interferon-γ left interneuron excitability unchanged and increased inhibitory postsynaptic currents frequency under voltage-dependent sodium channel blockage

**FIGURE 2:**
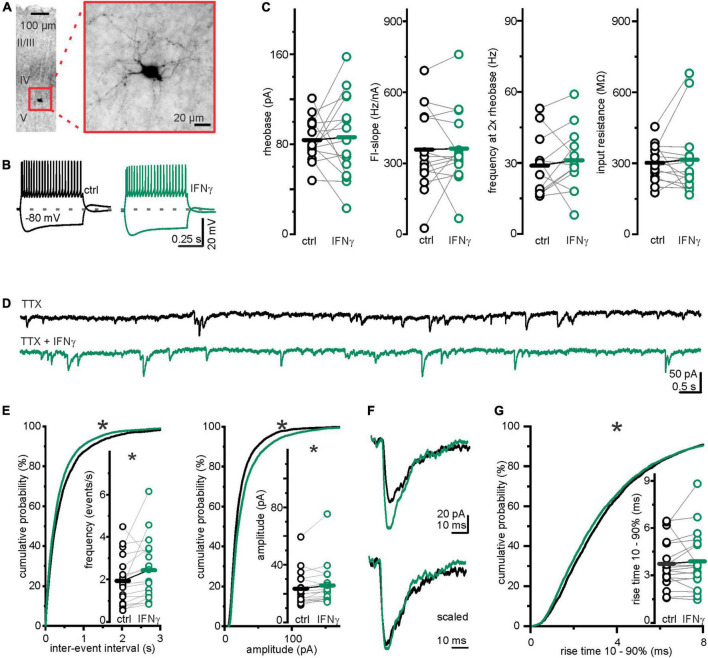
Changes in inhibitory transmission were not caused by interneuronal excitability alterations. **(A)** Example image of a *post hoc* biocytin staining, confirming layer 5 localization and interneuron morphology. **(B)** Example interneuronal voltage traces upon rectangular current injections before (*black*) and under (*green*) 25 min of continuous bath application of IFN-γ. **(C)** IFN-γ did not consistently affect interneuron excitability. Averages of excitability parameters: rheobase, FI-slope, firing frequency at 2 × rheobase, and input resistance remained comparable after 20–30 min of continuous application of IFN-γ. **(D)** Traces of miniature IPSCs (mIPSCs) recorded from layer 5 pyramidal neurons before (*black*) and with (*green*) 24.6 ± 1.0 min of continuous bath application of IFN-γ. Continuous bath application of tetrodotoxin (1 μM) suppressed action potential derived IPSCs. **(E)** Frequency and amplitude of mIPSCs were significantly increased following continuous bath application of IFN-γ for 24.6 ± 1.0 min. Interevent interval (IEI) probability plot is shifted to shorter IEIs upon IFN-γ (*p* < 0.0001, Kolmogorov–Smirnov test, ctrl = 4,139 IEIs vs. IFN-γ = 5,221 IEIs, 18 recordings, *N* = 8), amplitude probability is shifted to larger amplitudes (*p* < 0.0001, Kolmogorov–Smirnov test, ctrl = 4,175 events vs. IFN-γ = 5,257 events, 18 recordings, *N* = 8). **(F)** Exemplary mIPSCs recorded from the same neuron before (*black*) and under IFN-γ (*green*). The lower traces are peak-scaled for comparability. **(G)** IFN-γ shifted the rise time probability distribution of mIPSCs toward faster rise times (below 60%) (*p* = 0.001, Kolmogorov–Smirnov test, ctrl = 4,175 events vs. IFN-γ = 5,257 events, 18 recordings, *N* = 8). Mean rise time remained comparable (*inset*). For mIPSC recordings, *R*_*s*_ and *I*_*hold*_ remained comparable: *R*_*s ctrl*_ = 13.7 ± 1.7 MΩ vs. *R*_s IFN–γ_ = 13.8 ± 1.5 MΩ (*p* = 0.9, paired *t*-test, *n* = 18, *N* = 8); *I*_hold ctrl_ = −298.0 ± 36.0 pA vs. *I*_hold IFN–γ_ = −226.7 ± 22.5 pA (*p* = 0.09, paired Wilcoxon signed-rank test, *n* = 18, *N* = 8). *p < 0.05.

One possible presynaptic mechanism underlying the observed IFN-γ-induced increase in sIPSC frequency in early postnatal development includes augmented interneuron excitability. As medial ganglionic eminence and preoptic area interneurons have completed migration by the end of the first postnatal week (Bortone and Polleux, 2009), we performed current-clamp recordings in layer 5 interneurons that we identified by VGAT-Venus expression. For *post hoc* verification, we filled the neurons with biocytin and visualized them following the recording (Figure 2A). Bath applied IFN-γ left averaged parameters for sub- and suprathreshold excitability comparable, including: Input resistance, determining voltage change upon current injection, *R*_in ctrl_ = 302.0 ± 19.5 MΩ vs. *R*_in IFN–γ_ = 315.2 ± 39.2 MΩ, (*p* = 0.67, paired *t*-test, *n* = 15, *N* = 7), rheobase, lowest current capable of eliciting a single action potential, *I*_rheobase ctrl_ = 83.7 ± 4.9 pA vs. *I*_rheobase IFN–γ_ = 86.3 ± 9.7 pA (*p* = 0.79, paired *t*-test, *n* = 15, *N* = 7), slope of the frequency–current relationship *FI-slope*_ctrl_ = 358.0 ± 44.5 Hz nA^–1^ vs. *FI-slope*_IFN–γ_ = 362.2 ± 40.9 Hz nA^–1^ (*p* = 0.9, paired *t*-test, *n* = 15, *N* = 7) and frequency at 2 × rheobase *f*_2xRB ctrl_ = 28.8 ± 3.7 Hz vs. *f*_2xRB IFN–γ_ = 31.1 ± 4.0 Hz (*p* = 0.5, paired *t*-test, *n* = 12, *N* = 5). Notably, FI-slope and firing frequency at 2 × rheobase are parameters that reflect a neuron’s input–output relationship (Figures 2B,C).

Given the high variability and axonal divergence of GABAergic interneurons, we could not exclude subtype-specific IFN-γ action on interneuron excitability. We therefore next investigated whether the observed effect is dependent on presynaptic firing. Because IPSCs that might be elicited by presynaptic action potentials were not distinguishable from those resulting from spontaneous vesicle release by amplitude (Supplementary Figure 1D), we investigated the spontaneous vesicle release separately. We recorded miniature IPSCs (mIPSCs) from layer 5 pyramidal neurons in the presence of tetrodotoxin (TTX, 1 μM), a blocker of voltage-gated sodium channels. Despite continuously suppressed interneuronal firing, upon IFN-γ application mIPSC frequency increased from *f*_ctrl_ = 1.93 ± 0.27 events s^–1^ to *f*_IFN–γ_ = 2.43 ± 0.34 events s^–1^ (*p* = 0.04, paired *t*-test, *n* = 18, *N* = 8) and amplitude increased from *I*_ctrl_ = 23.5 ± 2.8 pA to *I*_IFN–γ_ = 25.7 ± 3.4 pA (*p* = 0.03, Wilcoxon signed-rank test, *n* = 18, *N* = 8) (Figures 2D,E). It remained possible that synapses of distinct interneuron groups (that connect to different parts of the neuron) are differentially affected by IFN-γ. Because the kinetics of synaptic currents, in particular the rise time (Xiang et al., 2002), give insights on synapse localization along the somatodendritic axis (e.g., perisomatic vs. dendritic site), we analyzed 10–90% rise times of mIPSCs before and after IFN-γ application and found that the mean rise time remained similar (rise time_ctrl_ = 3.7 ± 0.4 ms vs. rise time_IFN–γ_ = 3.9 ± 0.4 ms, *p* = 0.5, paired *t*-test, *n* = 18, *N* = 8). However, the cumulative probability distribution reveals a slight shift toward shorter rise times (Figures 2F,G). Because of the small yet significant difference between the probability distributions, we are not confident that this can be interpreted solely as an increased portion of perisomatic inhibition upon IFN-γ addition. A better distinction/a greater gap might be stashed by the relative electrotonic compactness of developing neurons.

Taken together, we have found that the IFN-γ-induced increase in sIPSC frequency appears independent of and cannot be attributed to an increase in layer 5 interneuron excitability or presynaptic firing.

### Protein kinase C inhibitor calphostin C, applied to the postsynaptic neuron, prevented interferon-γ mediated increase in mIPSC amplitude, but not frequency

**FIGURE 3:**
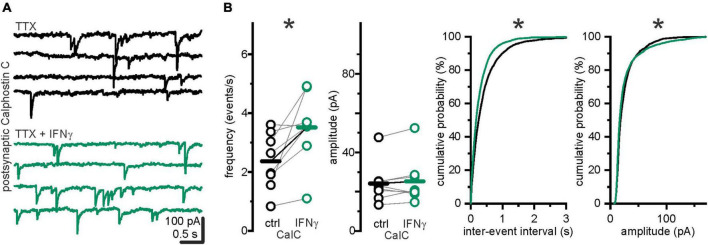
IFN-γ increased mIPSC frequencies when amplitude augmentation was suppressed by postsynaptic PKC inhibition *via* calphostin C. **(A)** Example traces of mIPSCs recorded under postsynaptic intracellular application of PKC inhibitor Calphostin C (100 nM) before (*black*) and with IFN-γ (*green*). **(B)** Under intracellular PKC inhibition, IFN-γ, bath applied for 25.0 ± 1.0 min, increased mIPSC frequency and shifted IEI probability distribution toward shorter IEIs (*p* < 0.0001, Kolmogorov–Smirnov test, ctrl = 2,254 IEIs vs. IFN-γ = 3,359 IEIs, 8 recordings, *N* = 2). Mean amplitudes remained comparable, however, amplitude probability distribution was slightly shifted toward lower amplitudes (below 80%), as well as higher amplitudes (above 90%) (*p* < 0.0001, Kolmogorov–Smirnov test, ctrl = 2,270 events vs. IFN-γ = 3,375 events, 8 recordings, *N* = 2). Series resistance slightly increased from *R*_*s* CalC ctrl_ = 15.4 ± 1.9 MΩ to *R*_*s* CalC IFN_–γ_ = 17.5 ± 2.2 MΩ (*p* = 0.001, paired *t*-test, *n* = 8, *N* = 2), holding current slightly decreased from −196.9 ± 27.1 pA to −121.1 ± 11.2 pA (*p* = 0.02, paired *t*-test, *n* = 8, *N* = 2). *p < 0.05.

We have previously reported that IFN-γ augments GABAergic currents by promoting postsynaptic membrane association of GABA_*A*_ receptors *via* a PKC-dependent mechanism (Janach et al., 2022) in late juvenile rats. To test whether the observed increase in s/mIPSC frequency is caused by higher amplitudes (i.e., whether more amplitudes are elevated above baseline noise levels), we recorded mIPSCs under postsynaptic block of PKC by Calphostin C (100 nM). Calphostin C in the postsynaptic neuron prevented an increase in mIPSC amplitude upon IFN-γ application (*I*_CalC ctrl_ = 24.1 ± 3.7 pA vs. *I*_CalC IFN_–γ_ = 25.3 ± 4.2, *p* = 0.4, Wilcoxon signed-rank test, *n* = 8, *N* = 2, Figure 3). However, mIPSC frequency increased from *f*_CalC ctrl_ = 2.36 ± 0.3 events s^–1^ to *f*_CalC IFN_–γ_ = 3.52 ± 0.42 events s^–1^ (*p* = 0.02, paired *t*-test, *n* = 8, *N* = 2).

These findings suggest that the observed IFN-γ-induced IPSC amplitude increase at the end of the first postnatal week is both postsynaptic and PKC mediated, as previously described for older stages (Janach et al., 2022). In addition, they indicate separate mechanisms for IPSC amplitude and frequency increase upon IFN-γ application. Thus, the persistence of the IFN-γ-induced increase in frequency under block of the major known postsynaptic mediator renders a postsynaptic contribution to the frequency effect unlikely.

### The interferon-γ-induced increase in sIPSC frequency was mimicked by nitric oxide and depended on inducible nitric oxide synthase and soluble guanylate cyclase

IFN-γ induces nitric oxide (NO) production (Blanchette et al., 2003). NO is also a known regulator of synaptic release (reviewed in Hardingham et al., 2013): NO donors increase (Li et al., 2004; Yang and Cox, 2007) and knockout of NO receptor soluble guanylate cyclase (sGC) decreases IPSC frequencies (Wang et al., 2017). Therefore, the IFN-γ induced elevation of IPSC frequency we observed in pyramidal neurons at P6–7 might be caused by elevated NO levels. Decreased IPSC frequencies upon NO, however, have been reported (Lee, 2009).

**FIGURE 4:**
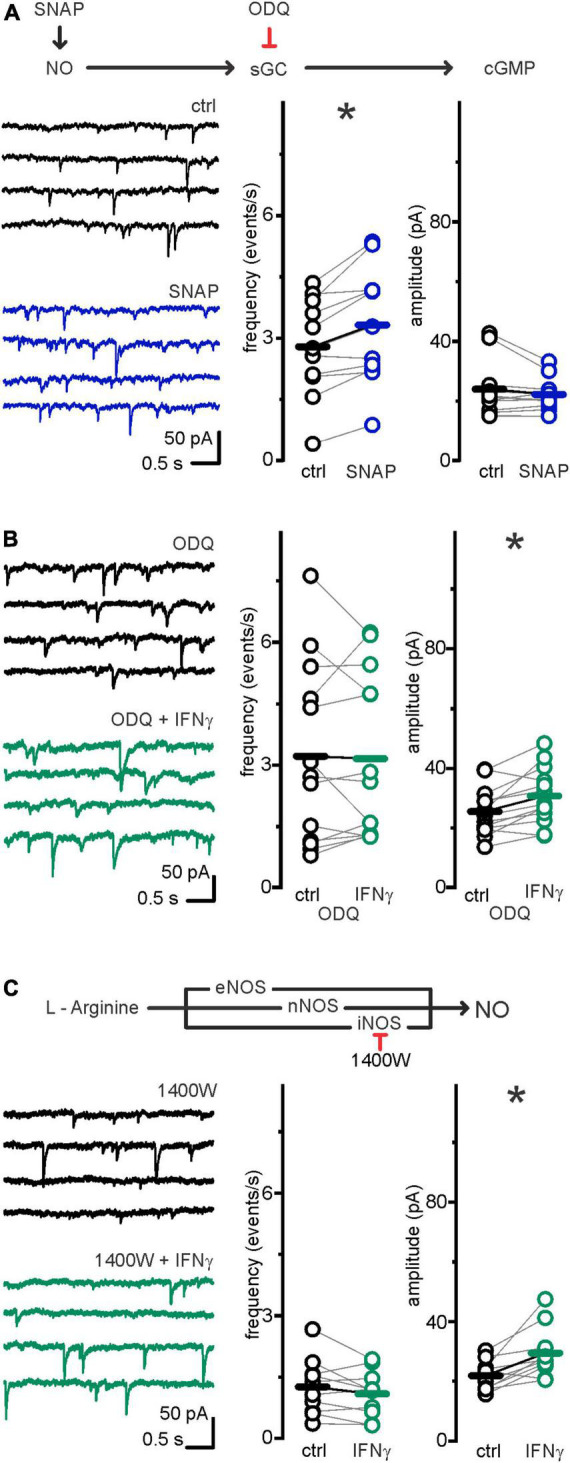
Increase in IPSC frequency, but not amplitude, depended on iNOS–NO–cGC–pathway. **(A)** *Upper row:* Drug interactions. SNAP spontaneously degrades, thereby releasing NO. ODQ selectively inhibits NO receptor soluble guanylate cyclase (sGC). *Lower row:* NO donor SNAP mimics IFN-γ effect on frequency but not amplitude: following continuous bath application of SNAP (300 μM) for 5 min, sIPSC frequency, but not amplitude, was significantly increased. Example traces left and grouped data right. Series resistance and holding current remained comparable: *R*_s ctrl_ = 12.5 ± 1.2 MΩ vs. *R*_s SNAP_ = 12.7 ± 1.2 MΩ (*p* = 0.65, paired *t*-test, *n* = 11, *N* = 3). *I*_hold ctrl_ = −265.8 ± 44.3 pA vs. *I*_hold SNAP_ = −262.9 ± 45.9 pA (*p* = 0.73, paired *t*-test, *n* = 11, *N* = 3). **(B)** sGC inhibitor ODQ (100 μM) inhibited sIPSC frequency, but not amplitude increase upon IFN-γ, bath applied for 23.1 ± 1.1 min. Example current traces left, grouped data right. Series resistance and holding current remained comparable: *R*_s ODQ ctrl_ = 12.1 ± 1.8 MΩ vs. *R*_s ODQ IFN–γ_ = 11.8 ± 1.5 MΩ (*p* = 0.6, paired *t*-test, *n* = 13, *N* = 7). *I*_hold ODQ ctrl_ = −365.8 ± 45.1 pA vs. *I*_hold ODQ IFN–γ_ = −306.3 ± 33.5 pA (*p* = 0.1, paired *t*-test, *n* = 13, *N* = 7). **(C)** NO is produced by three isoforms of nitric oxide synthase [inducible (iNOS), neuronal (nNOS), and endothelial (eNOS)]. In the presence of iNOS inhibitor 1400W (10 μM), IFN-γ, bath applied for 22.1 ± 0.8 min, increased sIPSC amplitude, but not frequency. Example current traces left, grouped data right. Series resistance and holding current remained comparable: *R*_*s* 1400W ctrl_ = 14 ± 1.7 MΩ vs. *R*_*s* 1400W IFN–γ_ = 13.6 ± 1.6 MΩ (*p* = 0.66, paired *t*-test, *n* = 10, *N* = 4). *I*_hold 1400*W* ctrl_ = −319.9 ± 34.6 pA vs. *I*_hold 1400 *W* IFN–γ_ = −308.4 ± 43.6 pA (*p* = 0.13, paired *t*-test, *n* = 10, *N* = 4). *p < 0.05.

To test whether NO is sufficient to induce an increase in inhibitory release at P6–7, we recorded sIPSCs and added SNAP (300 μM), an NO releasing agent widely used to study the impact of NO on neurophysiology (Li et al., 2004). Within 5 min, sIPSC frequency increased from *f*_ctrl_ = 2.79 ± 0.36 events s^–1^ to *f*_SNAP_ 3.32 ± 0.43 events s^–1^ (*p* = 0.002, paired *t*-test, *n* = 11, *N* = 3), while sIPSC amplitude remained comparable (*I*_ctrl_ = 24.0 ± 2.8 vs. *I*_SNAP_ = 22.2 ± 1.6 pA; *p* = 0.5, Wilcoxon signed-rank test, *n* = 11, *N* = 3; Figure 4A). NO donors can activate leaky, Cs^+^ sensitive, K^+^-channels (Kang et al., 2007). In our experiments, however, the Cs^+^ containing pipette solution should omit putative K^+^ currents. Accordingly, holding currents remained similar upon SNAP application: *I*_hold ctrl_ = −265.8 ± 44.3 pA vs. *I*_hold  SNAP_ = −262.9 ± 45.9 pA *p* = 0.73, paired *t*-test, *n* = 11, *N* = 3).

NO has been shown to impact GABAergic synaptic release *via* binding to its direct receptor sGC (Wang et al., 2017), and sGC is expressed in layer 5 neurons around P6–7 (Ding et al., 2005). If IFN-γ effects on presynaptic release are NO-mediated, blocking sGC should abolish them. Therefore, we tested this by utilizing ODQ (100 μM), a selective sGC blocker (Yang and Cox, 2007; Wang et al., 2017). Indeed, IFN-γ application failed to increase the sIPSC frequency in the presence of ODQ (*f*_ODQ ctrl_ = 3.22 ± 0.61 vs. *f*_ODQ IFN–γ_ = 3.16 ± 0.56 events s^–1^; *p* = 0.67, Wilcoxon signed-rank test, *n* = 13, *N* = 7). The IFN-γ induced increase in sIPSC amplitudes from *I*_ODQ ctrl_ = 25.5 ± 2.2 to *I*_ODQ IFN–γ_ = 30.8 ± 2.6 pA (*p* = 0.005, paired sample *t*-test, *n* = 13, *N* = 7), however, persisted under ODQ application (Figure 4B). This suggests that NO binding to sGC specifically mediates the increase in sIPSC frequency.

IFN-γ may induce NO production *via* inducible nitric oxide synthase (iNOS) (Stuehr and Marletta, 1987). To assess whether iNOS is involved in presynaptic IFN-γ effects, we recorded sIPSCs in neocortical pyramidal neurons in the presence of the potent and irreversible iNOS inhibitor 1400W (Garvey et al., 1997). We observed that the addition of 1400W (10 μM) reliably prevented sIPSC frequency increase upon IFN-γ application: Under 1400W, IFN-γ increased sIPSC amplitude (*I*_1400W ctrl_ = 21.8 ± 1.6 to *I*_1400W IFN–γ_ = 29.4 ± 2.7 pA; *p* = 0.007, paired *t*-test, *n* = 10, *N* = 3), whereas sIPSC frequency remained comparable (*f*_1400W ctrl_ = 1.26 ± 0.22 to *f*_1400W IFN–γ_ = 1.09 ± 0.18 events s^–1^, *p* = 0.16, paired *t*-test, *n* = 10, *N* = 3; Figure 4C). Of note, although 1400W may partly block neuronal (n)NOS (Garvey et al., 1997) with a nominal IC_50_ of 7.3 μM (Alderton et al., 2001), concentrations needed to effectively inhibit nNOS are ≥100 μM (Pigott et al., 2013).

In summary, these data indicate that IFN-γ increases presynaptic GABA release in an iNOS and sGC-dependent fashion.

### Interferon-γ facilitated short-term synaptic plasticity in GABAergic presynapses

**FIGURE 5:**
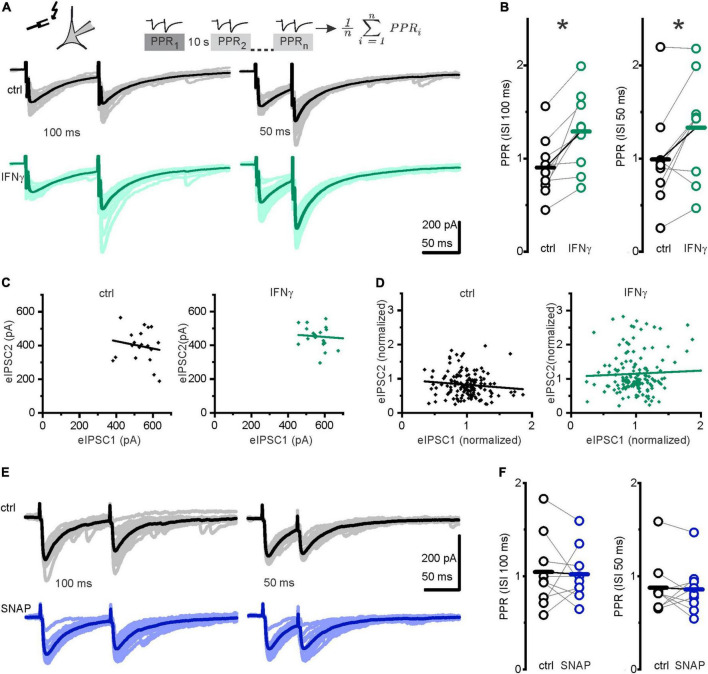
IFN-γ promoted paired-pulse facilitation. **(A)** *Upper row*: Paired-pulse paradigm: two consecutive IPSCs were evoked by electrical stimulation at 100 or 50 ms interstimulus interval (ISI). The paired-pulse ratio (PPR) was calculated by division of the second eIPSC amplitude by the first eIPSC amplitude. Twenty individual paired-pulse ratios were recorded and averaged for every data point. Stimulation was paused for 10 s between each pair. *Lower row:* Pairs of evoked IPSCs at 100 ms ISI (*left*) and 50 ms ISI (*right*), without (*black*) and with IFN-γ (*green*). Darker lines represent averages, stimulus artifacts truncated for clarity. Note comparable kinetics of evoked and spontaneous IPSCs of interfering sIPSCs during eIPSC decay. **(B)** IFN-γ, bath applied for 20 min, increased PPR (second amplitude divided by first amplitude). **(C,D)** PPR appears to be independent on previous release. **(C)** Plot of peak amplitudes of eIPSC2 vs. eIPSC1 for individual paired pulses from one neuron before (*ctrl, black*) and under 20 min of IFN-γ (*IFN-γ, green*). **(D)** Plot of normalized amplitude of eIPSC2 against eIPSC1 for all neurons investigated. Amplitudes were normalized to mean value of eIPSC1 for each pair. Lines represent linear regression [correlation coefficient −0.16 (*p* = 0.5) for ctrl and −0.08 (*p* = 0.7) for IFN-γ for one neuron **(C)** and −0.11 (*p* = 0.17) for ctrl and 0.04 (*p* = 0.6) for IFN-γ for all neurons **(D)**]. **(E)** Example traces of paired-pulse recordings before (*black*) and under (*blue*) application of the NO donor SNAP. Trace averages are shown in strong colors, and individual traces in light colors. **(F)** Paired-pulse ratios remained comparable after the application of SNAP (300 μM) for 5 min. Series resistance slightly increased from *R*_s ctrl_ = 11.1 ± 0.8 MΩ to *R*_s IFN–γ_ = 13.5 ± 1.0 MΩ (*p* = 0.01, paired *t*-test, *n* = 9, *N* = 6). Holding current remained grossly comparable *I*_hold ctrl_ = −316.7 ± 34.6 pA vs. *I*_hold IFN–γ_ = −252.4 ± 35.9 pA, *p* = 0.06, paired *t*-test, *n* = 9, *N* = 6). For SNAP experiments, series resistance and holding current remained comparable (*R*_*s* ctrl_ = 18.4 ± 2.5 MΩ vs. *R*_*s* SNAP_ = 19.8 ± 2.5 MΩ, *p* = 0.1, paired *t*-test, *n* = 9, *N* = 3; *I*_hold ctrl_ = −185.7 ± 36.0 pA vs. *I*_hold SNAP_ = −127.3 ± 13.6 pA). *p < 0.05.

Spontaneous and evoked GABA release neither completely overlaps nor is entirely separable (Horvath et al., 2020). To differentiate between IFN-γ effects on evoked vs. spontaneous GABA release, we used a paired-pulse paradigm revealing a paired-pulse ratio (PPR) that is commonly seen as an index of release probability (Kravchenko et al., 2006). We recorded eIPSCs evoked by pairs of stimuli at interstimulus intervals (ISI) of 100 or 50 ms. IFN-γ, bath applied for 20 min, consistently increased the PPR (defined as amplitude of the second response divided by the amplitude of the first response) at both intervals tested (from *PPR100ms*_ctrl_ = 0.9 ± 0.1 to *PPR100ms*_IFN–γ_ = 1.3 ± 0.1, *p* = 0.005, paired *t*-test, *n* = 9, *N* = 6 and from *PPR50ms*_ctrl_ = 1.0 ± 0.2 to *PPR50ms*_IFN–γ_ = 1.3 ± 0.2, *p* = 0.02, paired *t*-test, *n* = 9, *N* = 6; Figures 5A,B), i.e., it shifted the PPR toward facilitation. To estimate whether the PPR depends on the initial eIPSC amplitude, we used linear regression analysis in plots of second eIPSC amplitude vs. first eIPSC amplitude. Under control conditions, correlation coefficients (Pearson’s r) ranged from −0.6 to 0.1, none of these were statistically significant. Under IFN-γ application, correlation coefficients ranged from −0.67 to 0.45, one point of which was statistically significant at *r* = −0.46, *p* = 0.04. Using normalized amplitudes, we then pooled all experiments in each group. Pooled analysis showed no correlation between first and second eIPSC amplitudes (r_ctrl_ = −0.11, *p* = 0.17; r_IFN–γ_ = 0.04, *p* = 0.6, Figures 5C,D). This implies that PPR under control conditions and under the influence of IFN-γ does not depend on previous release as is seen in more mature rat neocortical pyramidal neurons (Supplementary Figures 2F,G; Xiang et al., 2002).

Because we have here investigated short-term plasticity (i.e., the ratio of consecutive eIPSC amplitudes at a given time point), we slightly released the restrictions on R_*s*_ for this particular series (see section “Materials and methods”). Subsequently, only four recordings matched our usual R_*s*_ constraints for absolute amplitude comparison in long-term recordings. Amplitudes remained comparable in a preliminary analysis of absolute amplitude values of evoked potentials in these (Supplementary Figure 2D). This is in contrast to the amplitude increase both in s/mIPSCs evinced in this study and in eIPSCs of more mature neurons studied previously (Janach et al., 2020). Moreover, while the release probability for s/mIPSC increased, the one for eIPSC probably decreased upon IFN-γ addition, thereby masking the (postsynaptic) IFN-γ effects. Given this, as well as the lack of PPD under control conditions, evoked release might be constrained in immature GABAergic synapses.

To test whether NO might contribute to IFN-γ-induced paired-pulse facilitation, we recorded another series using the paired-pulse paradigm and observed whether NO donor SNAP has an effect on PPR. SNAP (300 μM), applied for 5 min, did not alter the PPR at 100 or 50 ms ISI (*PPR100ms*_ctrl_ = 1.0 ± 0.1 vs. *PPR100ms*_SNAP_ = 1.0 ± 0.1, *p* = 0.8, paired *t*-test, *n* = 9, *N* = 3 and *PPR50ms*_ctrl_ = 0.9 ± 0.1 vs. *PPR50ms*_SNAP_ = 0.9 ± 0.1, *p* = 0.8, paired *t*-test, *n* = 9, *N* = 3; Figures 5E,F). As the PPR remained comparable under application of NO donor SNAP, we conclude that NO does not contribute to IFN-γ-mediated paired-pulse facilitation.

However, paired-pulse facilitation implies an increased capability of reliably transferring inhibition at higher frequencies (Luhmann et al., 2014). Following this, paired-pulse facilitation points to stronger inhibition at frequencies ≥10 Hz.

### Interferon-γ constrained excitability in the developing cortex

There is an ongoing debate about whether GABA acts inhibitory or excitatory at the early stages of development. At least in mice of the first postnatal week, GABA_*A*_R openings in L5 neurons lead predominantly to depolarizing currents (Rheims et al., 2008). This may drive specific patterns of activity in developing neuronal networks (Ben-Ari, 2002; Ben-Ari et al., 2007). However, hyperpolarizing GABA actions in the neocortex have also been seen in the first postnatal week (Daw et al., 2007). In any case, the opening of GABA_*A*_Rs, in addition to its effect on membrane voltage, mediates a drop in membrane resistance that shunts excitatory inputs (Staley and Mody, 1992). Therefore, even “depolarizing” actions can (1) be considered as inhibitory, (2) inhibit neuronal output *in silico* (Morita et al., 2006), and (3) suppress network activity *in vivo* (Kirmse et al., 2015).

**FIGURE 6:**
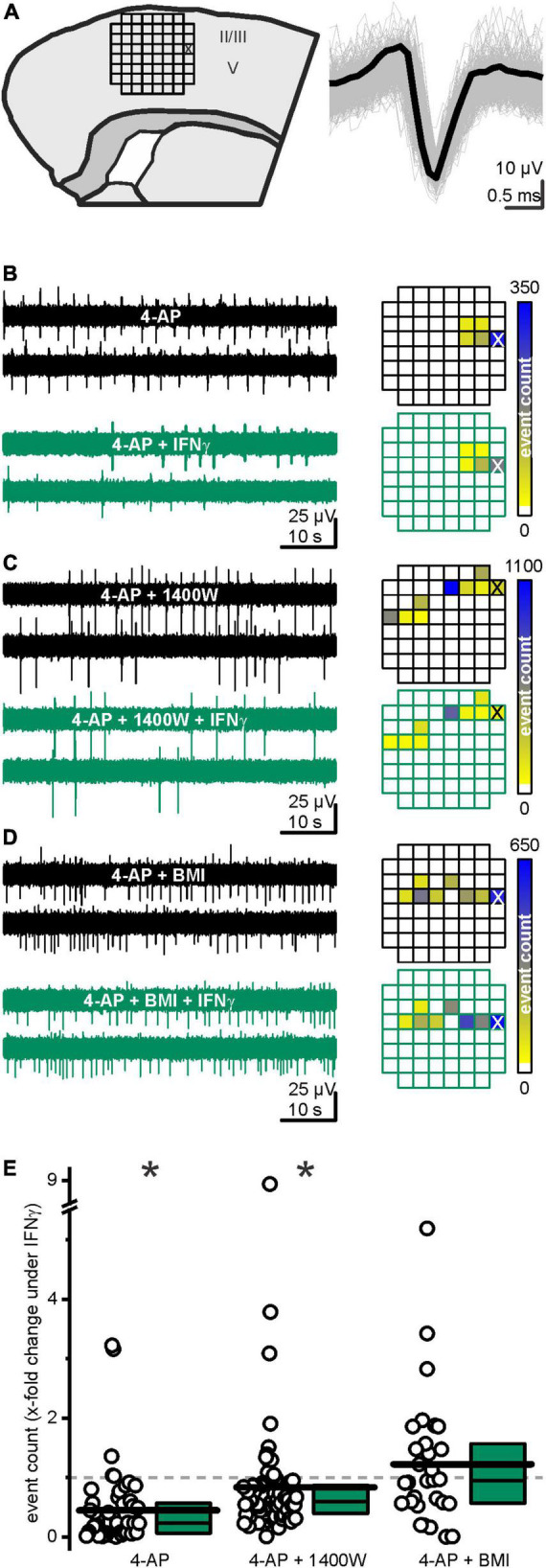
IFN-γ suppressed 4-aminopyridine (4-AP)-induced neuronal activity. **(A)** Experimental sketch: the grid represents the placement of the microelectrode array (MEA) (*left*). All accounts of one event, detected by a spike sorting algorithm, plotted on top of each other (*gray*) and the template they were matched to (*black, right*). All displayed events were recorded from the electrode marked with X in the figure on the left. **(B)** IFN-γ-curbed network activity in acute neocortical slices. Sample traces (*left*) recorded from the same electrode (marked with X in the heatmap below) before (*black*) and under (*green*) IFN-γ application. **(C)** Continuous iNOS inhibition *via* 1400W (10 μM) partly prevented IFN-γ-mediated network activity suppression. **(D)** IFN-γ required functioning GABA_*A*_ receptors to curb network activity. Under continuous bicuculline methiodide (BMI, 10 μM) application, IFN-γ failed to significantly alter event count. Example traces on top and exemplary heatmap below. **(E)** Relative event count change upon IFN-γ application in the absence of blockers and with 1400W or BMI, respectively. Most events considerably decreased in frequency upon IFN-γ. This attenuation is partly prevented in presence of 1400W and absent in the presence of BMI. Top, middle, and bottom lines of boxes represent percentiles 75, 50, and 25, respectively, and long bars represent means. *p < 0.05.

We here aimed at elucidating if and how the IFN-γ-induced increase in GABAergic transmission affects the developing cortical network. We therefore positioned brain slices on a microelectrode array (MEA, Figure 6A) and enhanced spontaneously occurring electrical events with 4-AP (50 μM). IFN-γ attenuated the frequency of these events on average by a factor of 0.45 ± 0.09 within 15 min (from *f*_ctrl_ = 22.6 ± 4.3 events min^–1^ to *f*_IFN–γ_ = 9.4 ± 2.8 events min^–1^, *p* < 0.0001, paired Wilcoxon signed-rank test, *n* = 49 distinguishable events, recorded in 8 slices from 6 animals, Figures 6B,E). This effect is GABA-mediated, as the frequency is not diminished upon IFN-γ when GABA_*A*_ receptors are blocked with bicuculline (10 μM) (average factor 1.2 ± 0.2, *f*_BMI_ = 17.8 ± 4.8 events min^–1^ vs. *f*_BMI IFN–γ_ = 10.9 ± 2.9 events min^–1^, *p* = 0.5, paired Wilcoxon signed-rank test, *n* = 30 distinguishable events, recorded in 5 slices from 3 animals, Figures 6D,E). Because our patch clamp recordings imply that NO elevated spontaneous GABA release upon IFN-γ application, we next tested if IFN-γ attenuates network activity when NO production was inhibited. In the presence of 1400W (10 μM), IFN-γ attenuated the frequency of events on average by a factor of 0.84 ± 0.13 (*f*_1400W_ = 48.3 ± 10.6 events min^–1^ vs. *f*_1400W IFN–γ_ = 31.1 ± 5.8 events min^–1^, *p* < 0.0001, Wilcoxon signed-rank test, *n* = 77 distinguishable events, recorded in 8 slices from 3 animals Figures 6C,E). However, 1400W significantly reduces the effect size (*p* < 0.0001, Kruskal–Wallis ANOVA, performed on relative change factors Figure 6E). Note that the absolute event numbers largely depended on experimental conditions of each individual slice, thus enabling only longitudinal paired comparison. The Ca^2+^-dependent stimulation of transmitter release by 4-AP applies to both glutamatergic and GABAergic synapses: however, the effect of 4-AP enhanced release of GABA might be countered by the inhibition of postsynaptic GABA currents (Gu et al., 2004), thereby enabling the overall pro-excitatory effect of 4-AP. Both effects theoretically could mask the sIPSC enhancement by IFN-γ. However, this would lead to an underestimation of the effect.

In summary, IFN-γ attenuates neocortical activity, i.e., it exerts network inhibition by constraining the occurrence of spontaneous events in a partly NO-dependent manner.

## Discussion

Combining measurements at the single cell and network level, we herein have found that, during a period of GABA-dependent cortical maturation, IFN-γ acutely increases the frequency and amplitude of GABAergic IPSCs on neocortical pyramidal neurons. This effect was not reversible within 30 min. The increase in IPSC frequency is not a result of enhanced interneuron excitability, since interneuronal sub- and suprathreshold excitability parameters remained stable, and elevated IPSC frequency was still present under action potential suppression. We have identified iNOS and sGC as mediators of increased IPSC frequency upon IFN-γ, plausibly acting through elevated levels of NO. Notably, manipulations of the iNOS–NO–sGC pathway revealed a differential effect on frequency and amplitude, i.e., a specificity of NO for the putatively pre-synaptically mediated frequency increase. Together with the apparent separation of the IFN-γ effects on the frequency and amplitude of IPSCs, the cessation of IPSC amplitude increases when PKC was blocked postsynaptically argues against an increased GABA_*A*_R sensitivity that might have uncovered subthreshold events from the noise. However, we cannot entirely exclude a contribution of synaptic modifications (i.e., an increased number of release sites) to the effect. Additionally, IFN-γ alters presynaptic function by shifting paired-pulse ratios toward facilitation. In contrast to the effect of IFN-γ on IPSCs, the effect on short-term synaptic plasticity was not dependent on NO. Functionally, IFN-γ constrains network activity in a GABA- and partially NO-dependent manner.

These new findings expand our existing knowledge of the neuromodulatory effects of IFN-γ on GABAergic transmission. The increase in s/mIPSC amplitude that we observed here resembles the one that we have previously observed in adult and late juvenile rats (Janach et al., 2020). There we identified PKC-dependent increase in postsynaptic GABA_*A*_ receptor number as an underlying mechanism mainly by peak-scaled non-stationary noise analysis (Janach et al., 2022). Non-stationary noise analysis, however, is difficult to apply in developing systems because it requires many low rise time IPSCs, but immature synaptic integration results in comparatively low IPSC frequencies. We have here found, however, that PKC inhibitor Calphostin C, when added to the postsynaptic neuron, prevented increased amplitudes upon IFN-γ addition. This, in addition to a comparable effect size, led us to conclude that the mechanism for amplitude increase resembles the one we found in more mature neurons. Therefore, we did not attempt to investigate whether NO-mediated postsynaptic mechanisms, such as PKG-dependent phosphorylation (McDonald and Moss, 1994; Robello et al., 1996), or whether the altered expression of GABA receptor subunits and Cl^–^ co-transporters that were reported in early life inflammation (Reid et al., 2013) contribute to, or counteract with, the s/mIPSC amplitude increases.

Instead, we have here focused on a developmental peculiarity in the neocortex of P6–7 rats, namely, the IFN-γ induced increase in IPSC frequency. Although, in retrospect, a trend toward an increase in IPSC frequency might become apparent in more mature animals (Janach et al., 2020, 2022), this was rather inconsistent. Why does IFN-γ cause a robust and reliable increase in IPSC frequency early postnatal but much less later? There are several plausible explanations. (1) Active heterodimers of the sGC (that we herein have recognized as necessary for the IPSC frequency increase) are more prevalent in young rats than in adult rats (Haase et al., 2010). (2) As microglia in early brain development are in an activated state and gradually inactivate by P10 (Hristova et al., 2010), they constitutively express iNOS (Crain et al., 2013) that enables mechanisms to rapidly increase NO other than *via de novo* iNOS synthesis (that takes 12 h; Jiang et al., 2022). Such mechanisms include both substrate and cofactor availability (for review, see Cinelli et al., 2020). This scenario is in line with the rather long IFN-γ exposure necessary for increased IPSC frequencies in hippocampal neurons of more mature animals (Brask et al., 2004). (3) Functional synapses on pyramidal cells in early development may originate from interneurons disparate to those seen at later stages. For instance, certain early generated somatostatin expressing interneurons are more likely to form functional synapses with pyramidal neurons around P5–7 in mice (Wang et al., 2019), and the presynaptic response to IFN-γ might be subtype-specific. However, our analysis of mIPSC rise times might suggest a slight preference for perisomatic synapses in the IFN-γ effect on spontaneous release probability.

In line with our pharmacological evidence that iNOS is involved in IFN-γ-induced frequency change, NOS blockage has been shown to prevent an IFN-γ-induced IPSC frequency increase (Brask et al., 2004) and IFN-γ slows γ-oscillations in an iNOS-dependent manner in organotypic hippocampal culture (Ta et al., 2019). In contrast to the tightly regulated, Ca^2+^-dependent, precise, and short-ranged nNOS action (Hardingham et al., 2013), iNOS can produce large amounts of NO independent of Ca^2+^ (Cinelli et al., 2020), plausibly enabling widespread multisynaptic actions. Regardless of the actual source, NO has been shown to increase IPSC frequency not only in the cortex (Wang et al., 2017) but also in the thalamus (Yang and Cox, 2007) and in the paraventricular nucleus (Li et al., 2004). Our data indicate that sGC is a prerequisite for the IFN-γ effect, therefore favoring a scenario in which NO impacts GABA release *via* downstream sGC targets (for review Hardingham et al., 2013). One of these putative targets, the hyperpolarization-activated cyclic nucleotide-gated cation (HCN) channel is involved in enhancing GABA release (Kopp-Scheinpflug et al., 2015; Wang et al., 2017). HCN channels are present in cortical GABAergic boutons but their role may be more complex because blocking them also increases IPSC frequency (Cai et al., 2021). However, the PKC-mediated attenuation of HCN that we have previously seen upon type-I and II interferon application (Reetz et al., 2014; Janach et al., 2020), as well as direct activation of release machinery by PKC (Yawo, 1999; Xu et al., 2014), would be incompatible both with the lack of IPSC frequency increase when sGC was blocked (Figure 4B) and with findings in the central amygdala where PKCε suppressed GABA release (Bajo et al., 2008). Other feasible sGC targets that could influence GABA release include P/Q Ca^2+^ channels as identified in brainstem neurons (Tozer et al., 2012) and faster vesicle recycling (Micheva et al., 2003). Again, NO-related mechanisms upstream of sGC, such as directly influencing L-Type Ca^2+^ channels (Tozer et al., 2012) or nitrosylation of release machinery proteins (Palmer et al., 2008), are less likely involved here. Rather, sGC-independent mechanisms inhibited cortical P/Q Ca^2+^ channels (Petzold et al., 2008). Further, the block of α-amino-3-hydroxy-5-methyl-4-isoxazolepropionic acid (AMPA) and NMDA receptors throughout our experiments excluded a contribution of directly NO-modulated NMDA (Dawson et al., 1996) or IFN-γ-increased AMPA Ca^2+^ conductance (Mizuno et al., 2008). NO may still induce Ca^2+^ release from intracellular sources as seen in striatal neurons (Horn et al., 2002). Taken together, we cannot rule out NO-mediated increase in presynaptic Ca^2+^, a classical modulator of transmitter release. Moreover, NO (donors) may elevate IPSC frequency Ca^2+^ independently (Li et al., 2004).

A parsimonious explanation of the obvious differences in the IFN-γ effect on s/mIPSCs and eIPSCs would be differential regulation of spontaneous and evoked GABA release by IFN-γ application indirectly implying segregation of the mechanisms underlying these two forms of vesicle release (Horvath et al., 2020). A plausible scenario for the peculiarities in evoked release would be that IFN-γ-mediated Ca^2+^ elevation might meet a relative lack of slow-binding Ca^2+^ buffer parvalbumin (PV) in the boutons of early postnatal interneurons (del Rio et al., 1994). The latter may decelerate the decay of residual Ca^2+^ (Caillard et al., 2000). This hypothesis awaits further experimental examination.

The relative lack of PPD combined with a large variance of PPRs evinced in our sample when compared with PPD obtained under the same conditions in more mature animals (Supplementary Figure 2F; see also Xiang et al., 2002), is an incidental but interesting finding that is possibly linked to inhibitory synapse development.

IFN-γ-mediated changes certainly interfere with the complex role GABA is playing in neocortical development. Although the actual outcome is hard to predict, a transient alteration in GABAergic (or glutamatergic) signaling during cortical development can lead to persisting changes (Kaindl et al., 2008; Stefovska et al., 2008). Balanced GABA is crucial for adequate neuronal stem cell proliferation, cell migration, and synaptogenesis (for review, Wang and Kriegstein, 2009). Increased GABAergic signaling enhances interneuron apoptosis, curbs network synchrony (Duan et al., 2020), and causes GABA synapse elimination (Wu et al., 2012). GABAergic interneurons control critical periods of synaptic plasticity in functional circuits of the cortex (for review Hensch, 2005), i.e., enhanced GABAergic transmission prematurely induces such periods (Fagiolini et al., 2004), leading to a mismatch of sensory stimuli and synaptic plasticity. In line with this, transient disturbances of interneuron generation and integration cause long-term behavioral abnormalities—even after cell counts normalize (Magno et al., 2021). On one hand, excitation–inhibition balance is stabilized by GABAergic currents adapting to activity (Xue et al., 2014) but also sensory deprivation may increase GABAergic inhibition (Maffei et al., 2006). On the other hand, in our study, GABAergic transmission induced by IFN-γ led to an overall reduced spontaneous activity, which, in a period of incomplete synaptogenesis, may lead to decreased excitatory synaptic integration (Burrone et al., 2002).

The neuroimmune interaction we describe here might have clinical implications as altered GABAergic transmission is associated with neurodevelopmental disorders (Deidda et al., 2014). Even in the absence of disease, transient pharmacological increase in GABAergic transmission in juvenile mice not only leads to anxiety-related behavior in adults (Shen et al., 2012) but also normalizes disturbed social preferences in IFN-γ deficient mice (Filiano et al., 2016). In ASD, IFN-γ is notably the most pronounced among commonly elevated pro-inflammatory cytokines (reviewed in Saghazadeh et al., 2019) and children with ASD have elevated nitrate plasma levels that correlate with levels of IFN-γ but not with other cytokines (Sweeten et al., 2004). Alcohol dependence increases IFN-γ production in brain immune cells, as well as mIPSC frequency in the central amygdala that normalizes upon application of the anti-inflammatory cytokine interleukin 10 (Patel et al., 2021).

Our findings shed light on the acute effects of IFN-γ on early GABAergic transmission thus providing a mechanism for how early inflammation could interfere with cortical development. This might have far-reaching consequences because functional disturbances in early microcircuits lead to aberrant cortical wiring at later stages that may cause neurological or psychiatric conditions (Molnár et al., 2020).

## Data availability statement

The original contributions presented in this study are included in the article/Supplementary material, further inquiries can be directed to the corresponding author.

## Ethics statement

The animal study was reviewed and approved by the European Communities Council Directive of September 22nd, 2010 (2010/63/EU) under the licenses T 0212/14, T-CH 0034/20 for wildtype rats and T0215/11 for transgenic rats.

## Author contributions

ND and US conceptualized the study and wrote the manuscript. ND performed and analyzed patch clamp recordings and *post hoc* stainings. AF supervised, analyzed, and contributed to MEA recordings. GJ, EB, and US contributed to patch clamp recordings. All authors contributed to the article and approved the submitted version.

## Footnotes

1
https://github.com/spyking-circus/spyking-circus

2
https://github.com/cortex-lab/phy
