# Supplementary material for: Interferon-γ augments GABA release in the developing neocortex via nitric oxide synthase/soluble guanylate cyclase and constrains network activity
Source: Front Cell Neurosci. 2022 Aug 10;16:913299. doi: 10.3389/fncel.2022.913299 (PMC9401097; doi:10.3389/fncel.2022.913299)
Supplement: Supplementary file 1 [file Data_Sheet_1.pdf]

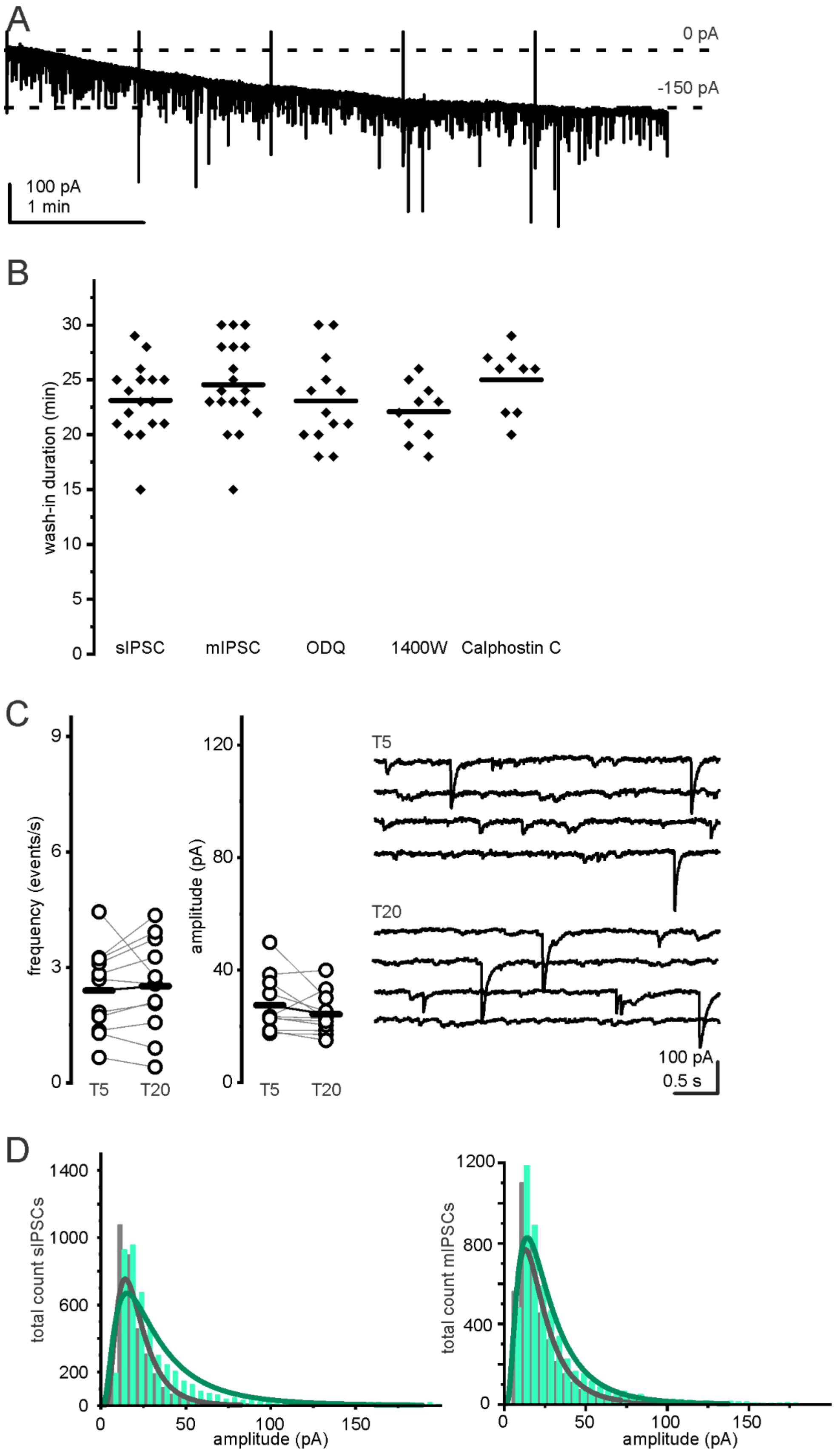
A
0 pA
-150 pA
100 pA
1 min
B
wash-in duration (min)
0
5
10
15
20
25
30
sIPSC
mIPSC
ODQ
1400W
Calphostin C
C
frequency (events/s)
0
3
6
9
T5
T20
amplitude (pA)
0
40
80
120
T5
T20
T5
T20
100 pA
0.5 s
D
total count sIPSCs
0
200
600
1000
1400
0
50
100
150
amplitude (pA)
total count mIPSCs
0
400
800
1200
0
50
100
150
amplitude (pA)

Supplementary Figure 1

A Exemplary recording showing typical current development (continuously clamped at –60 mV) after establishing whole-cell configuration using a CsCl based pipette solution. Note the slow increase in holding current from close to 0 pA to approximately –150 pA within five minutes, as intracellular solution exchanges with pipette solution. Artifacts that appear at the beginning of every minute are ± 3 mV test pulses that were used to monitor series resistance and capacitance.

B Overview over exposure times of IFN-γ for respective series.

C Recordings without IFN-γ show comparable sIPSC frequency and amplitude at minute 5 and minute 20. Note that the first 5 minutes were disregarded for all recordings to allow pipette solution and bath applied drugs to equilibrate. Series resistance and holding current remained comparable ($R_{s\,1}$ = 11.7 ± 1.1 MΩ *vs.* $R_{s\,2}$ = 12.1 ± 1.2 MΩ, P = 0.31, paired t-test. n = 11, N = 3; I_{hold1} = –353.9 ± 52.9 pA *vs.* I_{hold2} = –271.9 ± 43.44 pA, P = 0.06, paired t-test, n = 11, N = 3). The non-significant trend towards decrease in holding current might be a time-dependent effect (unlikely related to IFN-γ when occurring in other recordings).

D Amplitude histograms over all recorded events from Fig. 1C and Fig. 2E show no clear distinction between action potential evoked and spontaneous synaptic release by amplitudes before (grey/black) and under IFN-γ (light green/green).

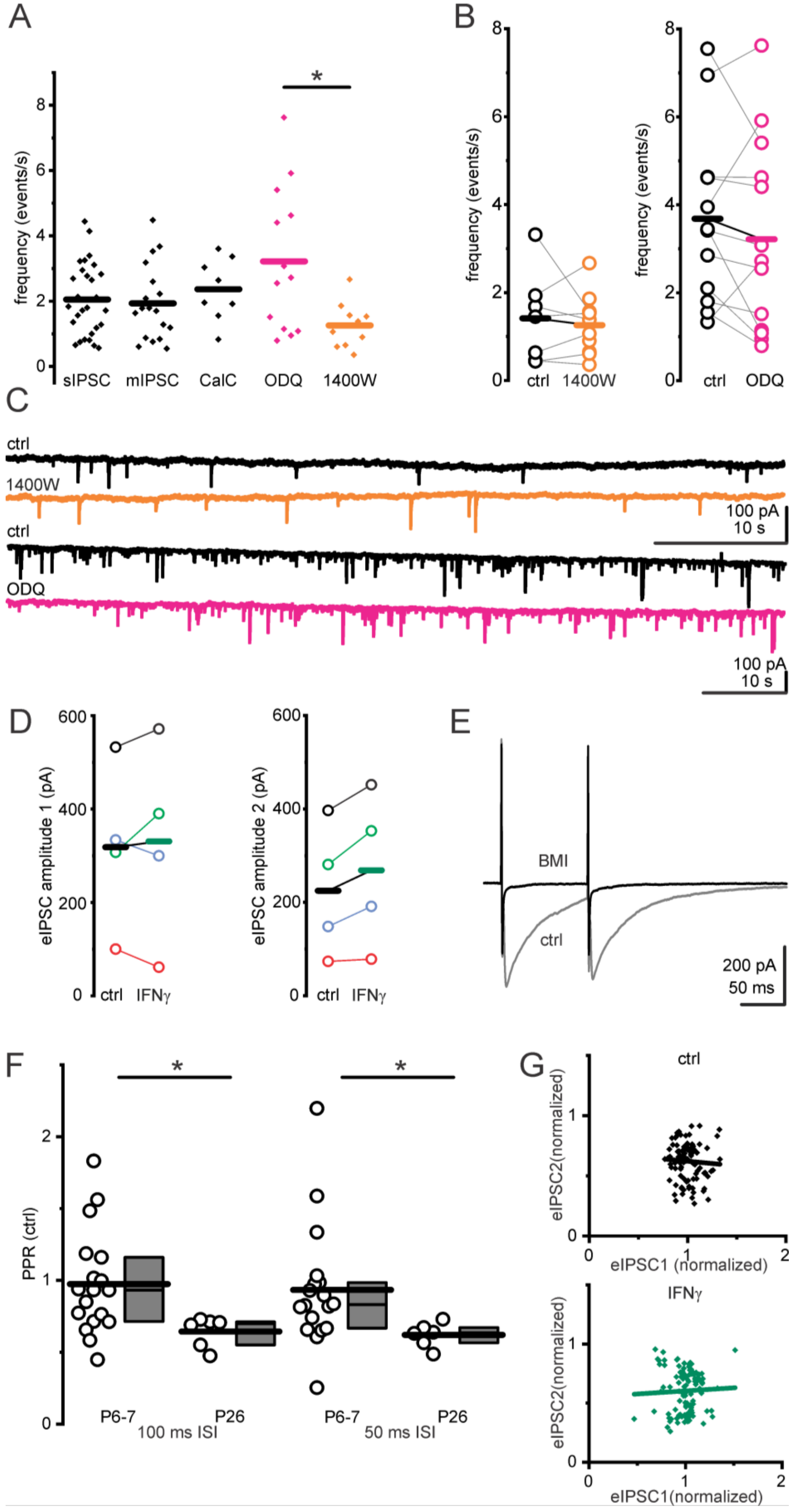
A
frequency (events/s)
sIPSC
mIPSC
CalC
ODQ
1400W
B
frequency (events/s)
ctrl
1400W
ODQ
C
ctrl
1400W
100 pA
10 s
ODQ
D
eIPSC amplitude 1 (pA)
eIPSC amplitude 2 (pA)
IFNγ
E
BMI
200 pA
50 ms
F
PPR (ctrl)
P6-7
P26
100 ms ISI
50 ms ISI
G
eIPSC2(normalized)
eIPSC1 (normalized)

Supplementary Figure 2

A Initial IPSC frequencies before application of IFN-γ show sizeable variance. ODQ (magenta) and 1400W (orange) means differ significantly (f_{1400W} = 1.26 ± 0.22 events s^{-1} *vs.* f_{ODQ} = 3.22 ± 0.61 events s^{-1}, P = 0.007, Bonferroni corrected one-way ANOVA).

B Pairwise analysis reveals that differences between initial frequencies in 1400W (orange) and ODQ (magenta) series cannot be attributed to the inhibitors ($f_{\mathbf{ctrl}\ (1400W)}$ = 1.41 ± 0.39 0.22 events s^{-1} *vs.* f_{1400W} = 1.31 ± 0.29 events s^{-1}, P = 0.7, paired t-test, n = 7; $f_{ctrl\ (ODQ)}$ = 3.68 ± 0.58 events s^{-1} *vs.* 3.39 ± 0.64 events s^{-1}, P = 0.4, paired t-test, n = 12). Note that means and sample numbers differ slightly from those given in the manuscript. Some recordings were started with respective inhibitors in the perfusate from the beginning and could not be considered here.

C Example traces from both series before (black) and with respective inhibitors (1400W, orange and ODQ, magenta).

D First and second evoked IPSCs that meet our inclusion criteria for amplitude comparison (R_s change below 25 %) remain comparable before and under IFN-γ ($I_{1\ ctrl}$ = 318.4 ± 88.4 pA *vs.* $I_{1\ IFN\gamma}$ = 330 ± 106.1 pA, P = 0.7, paired t-test, n = 4 *left;* $I_{2\ ctrl}$ = 224.9 ± 71.6 pA *vs.* $I_{2\ IFN\gamma}$ = 268.6 ± 83.1 pA, P = 0.054, paired t-test, n = 4 *right*). Colors of circles and lines label individual neurons.

E Paired pulse recordings demonstrate that evoked currents were completely blocked using $GABA_A$ antagonist 10 mM bicuculline methiodide (BMI). Control in gray, BMI in black. Twenty successive recordings were averaged for both conditions.

F Comparing inhibitory paired pulse ratios (PPR) under control conditions from P6 - 7 rats with respective PPR late juvenile rats (average age 26.2 ± 1.1 days, referred to as P26 in figures) published in (Janach et al., 2022) recordings from P6 - 7 rats show significantly higher paired pulse ratios: $PPR100ms_{P6-7}$ = 1.0 ± 0.1 *vs.* $PPR10Hz_{P26}$ = 0.6 ± 0.0 (P = 0.002, unequal variances t-test, n = 18, 6 respectively) and $PPR50ms_{P6-7}$ = 0.9 ± 0.1 *vs.* $PPR20Hz_{P26}$ = 0.6 ± 0.0 (P = 0.01, Mann-Whitney test, n = 18, 6 respectively). Top, middle, and bottom lines of boxes represent percentiles 75, 50 and 25, respectively, long bars represent mean PPR.

G Published data from late juvenile (Janach et al., 2022) was used to test if amplitudes of the first and second eIPSC are correlated. Amplitudes were normalized and pooled. None of the individual experiments (both in control or IFN-γ conditions) displays a correlation between the amplitude of the first eIPSC and the second eIPSC.

Reference

Janach, G. M. S., Böhm, M., Döhne, N., Kim, H.-R., Rosário, M., and Strauss, U. (2022). Interferon-γ enhances neocortical synaptic inhibition by promoting membrane association and phosphorylation of GABAA receptors in a protein kinase C-dependent manner. *Brain. Behav. Immun.* 101, 153–164. doi:10.1016/j.bbi.2022.01.001.
